# Mie-enhanced microfocused Brillouin light scattering for full wave vector resolution of nanoscale spin waves

**DOI:** 10.1126/sciadv.ady8833

**Published:** 2025-10-31

**Authors:** Jakub Krčma, Ondřej Wojewoda, Martin Hrtoň, Jakub Holobrádek, Jon Ander Arregi, Jaganandha Panda, Ekaterina Pribytova, Michal Urbánek

**Affiliations:** ^1^ Institute of Physical Engineering, Brno University of Technology, Technická 2, Brno 616 69, Czech Republic.; ^2^ CEITEC BUT, Brno University of Technology, Purkyňova 123, Brno 612 00, Czech Republic.

## Abstract

Magnons, the quanta of spin waves, are magnetic excitations of matter spanning through the entire crystal’s Brillouin zone and covering a wide range of frequencies ranging from subgigahertz to terahertz. Magnons play a crucial role in many phenomena, such as the reduction of saturation magnetization with increasing temperature or the Bose-Einstein condensation. However, established experimental techniques cannot resolve magnons with wave vectors between 30 and 300 rad μm^−1^. We address this gap by tailoring the Brillouin light scattering process with dielectric periodic nanostripes hosting Mie resonances. This approach enables access to the previously unmeasurable wave vector range while providing at the same time full wave vector resolution, all within a tabletop setup. Filling this gap can stimulate further experimental investigations of the fundamental phenomena associated with magnons as well as applications in computational and microwave devices. In addition, the same methodology can be applied to other excitations of matter, such as phonons, opening up possibilities in, e.g., mechanobiological studies.

## Teaser

Mie-enhanced Brillouin light scattering can reveal unexpected phenomena at previously inaccessible wave vector ranges.

## INTRODUCTION

**Fig. 1.**
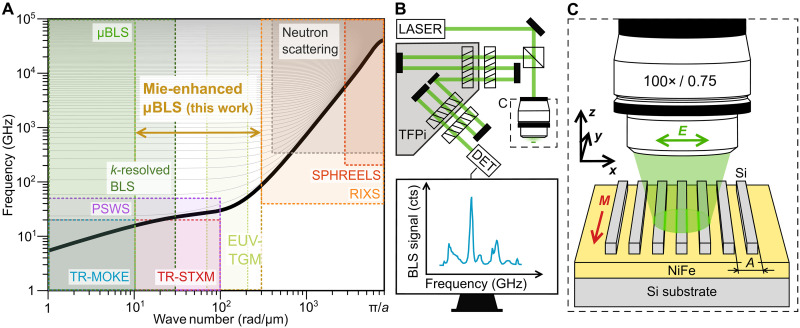
Comparison between experimental techniques, and schematics of Mie-enhanced BLS measurement. (**A**) Schematics of a spin wave dispersion relation in a permalloy thin film spanning over the whole Brillouin zone in an external magnetic field of 50 mT. Various experimental techniques are highlighted with their corresponding accessible frequency and wave number ranges. The range achieved using Mie-enhanced μBLS in this work is emphasized. (**B**) Experimental setup for μBLS measurements. Green laser light (532 nm) is guided and focused onto the sample through an objective lens ( NA=0.75 ). The backscattered light from the sample is collected by the same lens and analyzed using a tandem Fabry-Perot interferometer to measure the spin wave frequency spectrum. (**C**) Sample and measurement geometry. The sample consists of Si nanostripes arranged with a periodicity *A* on top of a permalloy 27.6-nm-thick film deposited onto a Si substrate.

The field of magnonics has emerged as a promising area of research, focusing on the collective magnetic excitations known as spin waves and their associated quasi-particles, magnons. Magnonics can be categorized into two areas of interest: mesoscopic magnonics and research of fundamental properties of matter at the atomic scale. The field of mesoscopic magnonics searches for energy efficient computation paradigms, devices, or studies interesting behavior of matter at micro- and nanoscale (*1*–*7*). These studies are usually performed on spin waves with their wave vectors spanning only from 0 to approximately 100 rad μm^−1^, and use variety of magneto-optical techniques such as *k*-resolved Brillouin light scattering (*k*-resolved BLS), microfocused BLS (μBLS), time-resolved magneto-optical Kerr effect (TR-MOKE), time-resolved scanning transmission x-ray microscopy (TR-STXM), nitrogen-vacancy microscopy (limited only to the very low frequencies and thus not relevant for the following discussion), or electrical techniques such as propagating spin-wave spectroscopy (PSWS) (*8*–*18*). In contrast, to investigate fundamental properties of matter, such as chiral splitting in altermagnets (*19*, *20*) or spin-orbital separation (*21*), one usually needs to investigate magnons across the whole Brillouin zone and at hundreds of gigahertz to terahertz frequencies. These characterization needs can fulfilled by inelastic neutron scattering, resonant inelastic x-ray scattering (RIXS), or spin-polarized high-resolution electron energy-loss spectroscopy (SPHREELS) (*22*–*25*). However, there is no single experimental technique that covers the whole range of interest, with each technique being confined to a particular wave vector and frequency range resulting from various technical and fundamental constraints (see Fig. 1A).

Concerning wave number, imaging techniques are limited by the probing spot size to approximately 10 rad μm^−1^ for μBLS and TR-MOKE and 100 rad μm^−1^ for TR-STXM (although the theoretical limit of the STXM is approximately 250 rad μm^−1^, it has never been experimentally demonstrated, most likely due to the problems with the low amplitude of the excited short-wavelength spin waves and insufficient signal sensitivity of the detection) (*10*, *26*). *K*-resolved BLS is limited by the conservation of momentum to approximately 30 rad μm^−1^ (*8*), whereas PSWS is restricted by the excitation efficiency to approximately 100 rad μm^−1^ (*27*–*29*). Regarding frequency, μBLS has no limitation in the range of interest as various types of spectrometers can be used, such as (tandem) Fabry-Perot interferometers (*30*), virtual phase arrays (*31*), or grating monochromators (*32*). Contrary to that, techniques that rely on coherent excitation (pump-probe, electrical detection) are limited by the used electronics, typically to tens of gigahertz. Techniques used to measure spin waves across the whole Brillouin zone (inelastic neutron scattering, SPHREELS, and RIXS) are limited in resolving lower wave numbers and frequencies by their resolution (typically over 300 rad μm^−1^ and 100 GHz).

In between these two areas, a relevant experimental blind spot becomes apparent, which spans approximately from 100 to 300 rad μm^−1^ for low frequencies, and from 30 to 300 rad μm^−1^ for high frequencies (see Fig. 1A). Various attempts to fill this gap were made by using near-field enhancement of the μBLS signal, which can lift the restriction posed by the conservation of momentum law by introducing an imaginary part to the momentum of the incident electromagnetic wave. This principle was used to enhance the accessible wave number range by using plasmon resonances; however, in practical applications, the signal yield is very low (*33*–*35*). Recently, we have reported that dielectric nanoresonators hosting Mie resonances enhance both the wave vector sensitivity and the signal yield even for phase-resolved BLS measurements (*36*, *37*). However, without coherent excitation, individual nanoresonators lack wave vector resolution and thus can only provide limited information about the studied excitation. Note, that recently, a new technique using extreme ultraviolet transient magnetization gratings (EUV-TMG) has been introduced that allows the study of short-wavelength spin waves (*38*). However, this technique is limited only to materials with accessible absorption edges in the used energy range and can only study spin waves excited by the femtosecond EUV pulses. Moreover, the spatial resolution is limited to hundreds of micrometers.

Here, we address these limitations by introducing a periodic array of dielectric nanostripes on top of the magnetic system of interest (a NiFe film) and measuring it in a standard μBLS setup. In this configuration, the light’s wave vector is superimposed with the lattice vector of the nanostripe array. The concept draws inspiration from wave propagation in periodic potentials, such as spin waves in artificial magnonic crystals (*39*, *40*). However, since the nanostripes are nonmagnetic, they do not modify the dispersion relation, allowing us to probe spin waves in an otherwise unaltered material system (*41*). As a result, we achieve full wave vector resolution, both magnitude and direction, across a previously inaccessible *k*-vector range, a capability not attainable with individual dielectric nanoresonators (*36*, *37*). This approach goes far beyond the experiments from the previous studies, as we now show that it is even possible to resolve wave vectors of incoherent magnons (e.g., thermally excited or parametrically pumped).

## RESULTS AND DISCUSSION

To measure inelastically scattered spectra, we use a standard BLS setup (see Fig. 1B). The laser light is focused by a high–numerical aperture (*NA*) objective lens ( NA=0.75 ) onto a 440-nm spot on the sample (*36*). The backscattered light is then collected by the objective lens and analyzed by the multipass tandem Fabry-Perot interferometer, which measures the spin wave frequency spectrum (*30*).

The sample consists of a (27.6 ± 0.1) nm-thick permalloy film deposited on a Si substrate (for thickness measurement details see note S1). Periodic Si nanostripes, acting as Mie resonators, were fabricated on top of the magnetic film by electron beam lithography, ion beam sputtering, and a lift-off process. The stripes form gratings with varying periodicities, each grating covering 6.5 × 6.5 μm^2^ of the sample, see Fig. 1C.

**Fig. 2.**
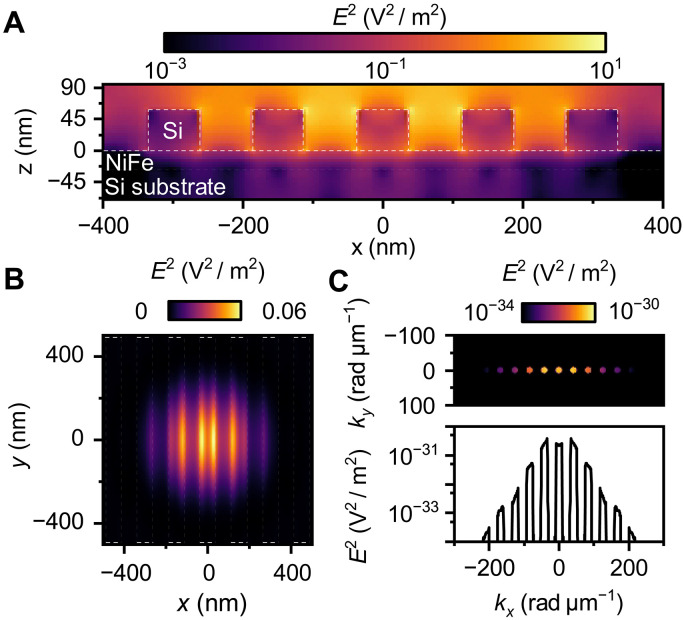
FDTD simulations of the electric field distribution of the incident laser light on the magnetic layer. (**A**) Logarithmic-scale electric field distribution in the *x,z*-plane, showing a cross-sectional view of the dielectric grating. Strong electric field localization is observed at the edges of the stripes (hot spots). White dotted lines indicate the interfaces. (**B**) Electric field distribution in the *x,y*-plane at the middle of the permalloy layer. Periodic field modulation is visible within the Gaussian beam spot. White dotted lines indicate the stripe positions. (**C**) Reciprocal electric field distribution in the *x,y*-plane, obtained via Fourier transformation of the data from (B). The 1D plot (lower panel) shows the logarithmic intensity profile at $k_{y}=0$ , highlighting the field’s periodic distribution in the reciprocal space.

To describe the Mie-enhanced BLS process quantitatively, it is essential to understand how the incident light interacts with the dielectric nanoresonators and the magnetic material. When the driving incident electric field ( $E_{\text{dr}}$ ) interacts with the periodic dielectric nanostripes array with the lattice constant *A*, it generates a strongly localized electric field (hot spots) at the edges of the individual nanostripes within the illumination Gaussian spot (see Fig. 2A). These hot spots are the result of excitation of Mie resonances within the nanoresonators, which modulate the field distribution inside the magnetic layer (Fig. 2B). This modulation leads to a periodic electric field distribution, enabling the light to couple only to states with wave numbers corresponding to multiples of 2π/A , even beyond the free space light accessible boundaries given by momentum conservation (Fig. 2C) (*34*, *36*). At the same time, the high symmetry of periodic dielectric nanostripes in the *y* direction (see Fig. 2C) leads to a strong localization of the electric field along the $k_{y}$ direction in the reciprocal space. The widths of the k=0 peak and all other higher-order peaks are determined by the width of the illumination laser spot. This modulated electric field interacts inelastically with the dynamic magnetization (spin waves) via magneto-optical coupling, inducing polarization

$$P_{m}\left(r,\omega +\omega_{m}\right)=\chi_{m}\left(r,\omega_{m}\right)E_{\text{dr}}\left(r,\omega \right)$$ (1)

where $\chi_{m}\left(r,\omega_{m}\right)$ is the dynamic magnetic susceptibility tensor (*42*), $E_{\text{dr}}\left(r,\omega \right)$ the field generated in the layer by the incident laser beam (driving field), and $\omega_{m}$ the frequency of spin waves. Since the frequency of the incoming radiation is much larger than that of the spin waves, we omit its dependency on the induced polarization.

The polarization acts as a local source of radiation and its out-coupling from the magnetic layer and subsequent propagation toward the detector can be described using the dyadic Green’s function formalism (*10*). However, because of the broken spatial symmetry caused by the presence of the dielectric periodic nanostripes, the dyadic Green’s function is not known analytically and it needs to be derived from numerical simulations. Recalling that it represents the impulse response of the system to a point source, the task of finding the dyadic Green’s function amounts to running a large set of simulations, where the position of the point source is varied within a sufficient range and with an adequate resolution. Alternatively, one can switch to reciprocal space and attempt to reconstruct the angular spectrum representation of the dyadic Green’s function (*36*), i.e., an impulse response to a plane wave, but since the dielectric nanostripes do not have radial symmetry, the number of simulations to obtain the dyadic Green’s function with a sufficient precision becomes prohibitively high.

To overcome these limitations, we exploited the reciprocity theorem of electromagnetism, drawing an inspiration from a similar approach for the scattering process in scanning near-field optical microscopy (*43*). The reciprocity theorem simplifies the scattering problem by introducing the concept of a virtual source located at the detector and providing a clear mathematical link between the electric field generated by this virtual source and the radiation emitted by an actual source, later collected by the detector. In accordance with our BLS detection scheme, the virtual source is represented by an electric dipole $p_{v}$ oriented perpendicularly to the incident laser light. Denoting $E_{v}\left(r\right)$ the electric field generated by the virtual source within the magnetic layer and $E_{m}\left(r_{\text{det}}\right)$ the electric field produced by the polarization source $P_{m}\left(r\right)$ at the position of the detector $r_{\text{det}}$ , the reciprocity theorem enforces the following relation

$$p_{v}\left(r_{\text{det}}\right)\cdot E_{m}\left(r_{\text{det}}\right)=\int dr^{3}P_{m}\left(r\right)\cdot E_{v}\left(r\right)$$ (2)

where the integration spans, in principle, the entire magnetic layer, but in practice is limited to the illumination spot. Assuming that the induced polarization has the form given by Eq. 1 and that only a single magnon with a lateral wave vector $k_{m}⊥z$ is being excited (for details, see Materials and Methods), the detected BLS signal can be expressed as

$$\begin{array}{c} \sigma \left(k_{m},\omega_{m}\right)∼{\left|p_{v}\left(r_{\text{det}}\right)\cdot E_{m}\left(r_{\text{det}}\right)\right|}^{2}=\\ ={\left|\sum_{i,j}\int dz\chi_{m}^{\mathrm{ij}}\left(z,\omega_{m}\right)T_{\mathrm{ij}}(k_{m},z)\right|}^{2} \end{array}$$ (3)

where the transfer function $T_{\mathrm{ij}}\left(k_{m},z\right)$ defined as

$$T_{\mathrm{ij}}\left(k_{m},z\right)=\int dr_{∥}^{2}E_{v}^{i}\left(r\right)E_{\text{dr}}^{j}\left(r\right)e^{ik_{m}\cdot r_{∥}}$$ (4)

effectively determines the range of spin wave wave vectors that the system is able to couple with, regardless of whether these spin wave states are occupied or not. Note that the vector nature of the electric fields makes the transfer function a rank two tensor. Furthermore, the result of the integration over the vertical coordinate *z* will generally depend on the type of magnon that is being excited, but given the relatively large thickness of our metallic layer with respect to the penetration depth of the electric field, so that it effectively probes only the top portion of the magnetic layer, we simplified our analysis by recording the field and evaluating the transfer function only within a single plane.

As Eq. 3 suggests, the problem is reduced to finding the driving field ( $E_{\text{dr}}$ ), the virtual field ( $E_{v}$ ), and the dynamic magnetic susceptibility ( $\chi_{m}$ ). The electric fields are obtained by performing two finite-difference time-domain (FDTD) simulations with polarizations perpendicular to each other, and the dynamic susceptibility is obtained by a semi-analytical approach assuming only the first-order magneto-optical coupling (*10*).

**Fig. 3.**
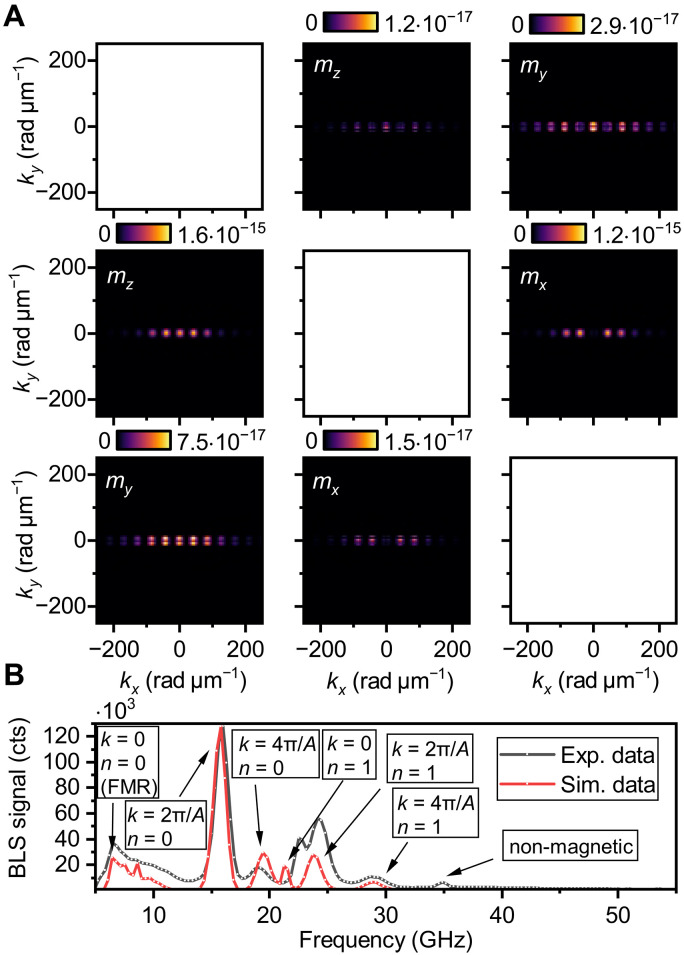
FDTD simulation data and results for a dielectric array with periodicity A=150  nm. (**A**) Transfer function tensor components, with each panel labeled by the corresponding dynamic magnetization component that it interacts with (assuming only linear magneto-optical coupling). The diagonal components are not computed, as they do not contribute to the final signal. (**B**) Simulated BLS spectra compared with experimental results. Peaks are annotated with their corresponding wave numbers and perpendicular standing spin wave (PSSW) modes.

We apply this model to obtain the transfer function of Si nanostripes with the periodicity A=150nm, with materials and geometry matching with the fabricated sample (see Fig. 3A). As expected, the transfer function shows maxima at the multiples of the periodicity, i.e., 2π/A=42 rad μm^−1^, 4π/A=84 rad μm^−1^. Also, these maxima show strong directionality, as they occur only for the close to zero *y*–wave vector component (along the long axis of the stripes), which is in the agreement with reciprocal distribution of the electric field, see Fig. 2C. This behavior can be exploited to gain directional and magnitude sensitivity in the BLS process.

The transfer matrix components $T_{\mathrm{xy}}$ and $T_{\mathrm{zy}}$ , which couple to the $m_{z}$ and $m_{x}$ magnetization components, respectively, are approximately two orders of magnitude larger than the remaining transfer matrix components. However, depending on the exact geometry of the incident electric field, the direction of the static magnetization, the coupling mechanism and the ellipticity of the spin-wave mode, the absolute contribution to the resulting signal can vary. For example, if the static magnetization is aligned along the *x* direction and only linear magneto-optical coupling is assumed, the contribution of the $T_{\mathrm{zy}}$ component of the transfer matrix function to the resulting signal would be zero.

The width of the illumination spot determines the wave vector resolution (the width of the periodic peaks in the transfer matrix components). The larger the illuminated area, the better wave number resolution can be achieved. In our experiment, we have used a 440-nm-wide Gaussian spot, resulting in a wave vector resolution of approximately 10 rad μm^−1^. The resolution is independent of the order of the peak of transfer function and of the periodicity of the nanostripe array. The increase in wave vector resolution can only be achieved at the expense of spatial resolution and vice versa.

In Fig. 3B, this transfer function has been used to model the BLS signal and compare it directly with the experiment. We can observe the formation of peaks at the frequencies given by the dispersion relation (see Fig. 1A) matching with *k*-vector sensitivity imposed by the periodicity of the dielectric nanostripes ( A=150 nm) (see Figs. 2C and 3A). The intensity of the μBLS accessible mode on a bare film ( k≲10 rad μm^−1^, n=0 ) is strongly suppressed compared to the peak with matching periodicity to the periodic dielectric nanostripes ( k=2π/A and n=0 ). The intensity of the matching higher-order peaks ( k=4π/A , k=6π/A , etc.) decays exponentially, so only the first two orders are visible in this representation. However, thanks to the high contrast of the tandem Fabry-Perot interferometer (150 dB), even very small contributions can be resolved experimentally. Also note that the process is robust and fabrication imperfections do not noticeably affect the resulting spectra; see micrographs of nanostripe arrays in the sample preparation section in Materials and Methods.

At higher frequencies, we can also observe peaks caused by the first-order perpendicular standing spin waves (PSSWs; n=1 ). There, we can see a small frequency offset between the model and the experimental data, which will be discussed later in the text.

**Fig. 4.**
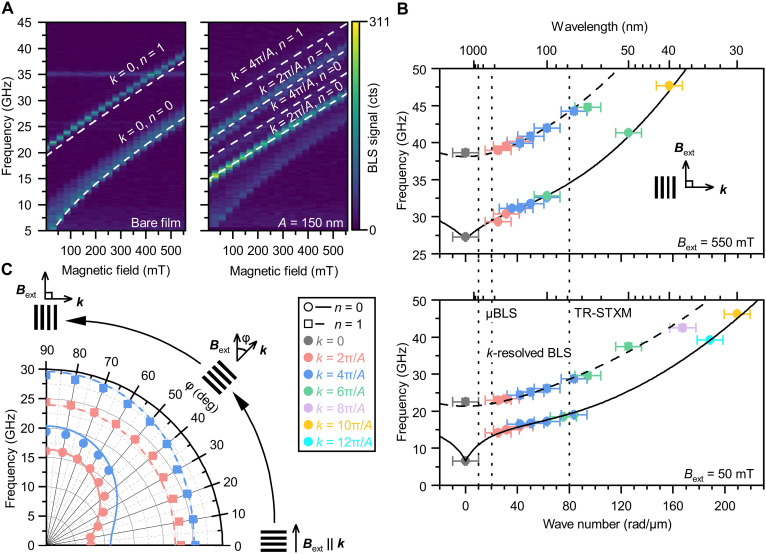
Experimental results measured on arrays with varying periodicity *A*. (**A**) Comparison of 2D BLS spectra (frequency versus magnetic field) for a bare magnetic film and a dielectric array. The presence of the array leads to additional accessible magnon wave numbers and their higher-order thickness modes. The curves represent theoretical calculations using the material constants obtained from (B). (**B**) Magnon dispersion relation at 550 mT (top panel) and 50 mT (bottom panel) obtained from arrays with periodicities ranging from 150 to 300 nm, showing discrete modes (data points) fitted with theoretical dispersion curves using the Kalinikos-Slavin model (*44*, *45*). The color code of the experimental points represents order of multiples of the periodicity of the analyzed peak. The periodicity of the nanostripe array can be deduced from its order and from the position of the point. (**C**) Angular dependence of the frequency of magnons with specific wave number on the angle between the external magnetic field and the magnon wave vector. The data were acquired on the grating with *A* = 150 nm. The polarization of the incident laser light was perpendicular to the magnetic field. The curves represent theoretical predictions calculated using the fitted material parameters from (B).

To verify the origin of the signal, we measured the BLS spectra on the same dielectric stripes ( A=150 nm) in different external magnetic fields and compared them with a standard μBLS measurement on the bare film (see Fig. 4A). On the bare film (Fig. 4A, left), only spin waves around the center of the Brillouin zone ( k=0 ) are visible for both PSSW modes. However, as mentioned before, with the periodic dielectric nanostripes, we resolve spin waves with wave numbers corresponding to the first two multiples of 2π/A . For both experiments, we can clearly observe the expected shift of the spin wave frequencies when increasing the magnetic field, which agrees well with the analytical formula for spin wave dispersion and does not change by the introduction of the nanostripe array (*44*, *45*). Also note, that the intensity of the modes up to fourth order is higher or comparable to the signal strength measured on the bare film.

To extract the dispersion relation of the NiFe film, we performed a similar experiment as in Fig. 3B. We used multiple nanostripe arrays with periodicities ranging from 150 to 300 nm and measured BLS signal in low (50 mT) and high (550 mT) magnetic fields. The arrays with different periodicities have a robust optical response across different wavelengths and their dimensions are limited only by nanofabrication capabilities (see note S2). Also, note that the measured wave vector of the spin waves is determined by the periodicity of the nanostripe array and is not influenced by the illumination light wavelength or *NA* of the used objective lens. By analyzing the measured peak positions, we were able to reconstruct the dispersion relation (Fig. 4B). Please note that if more peaks coincide in the frequency, we were not able to resolve them and we only associated its frequency position with the wave number with the highest expected signal; for details, see Materials and Methods. With just eight separate measurements, we were able to obtain a wide wave number range spanning from dipolar to exchange dominated spin waves. The experimentally obtained results were fitted with theoretical dispersion relations (*44*, *45*). The wide range of measured wave numbers allowed us to extract from a single fit the saturation magnetization $M_{s}=\left(700\;\pm \;20\right)$ kA/m, the gyromagnetic ratio γ/2π=(30.4±0.4) GHz/T, and also usually strongly correlated material parameters, the exchange constant $A_{\text{ex}}=\left(8.1\;\pm \;0.3\right)$ pJ/m and the film thickness t=(26.0±0.3) nm. However, the model has a frequency offset (approximately 1.5 GHz) from the experimental data for modes n=1 and k=0 . This may be due to more complicated boundary conditions than totally unpinned spins used in the modeled dispersion relation.

To demonstrate the directional sensitivity of the BLS process on periodic dielectric nanostripes, we rotated the sample along the *z* axis while keeping the direction of the external magnetic field and the incident electric field constant, and acquired the azimuthal dependence of BLS spectra. Because of the induced periodicity of the electric field in the direction of the periodic nanostripes, the direction of the detected wave vector followed the angle of the rotation of the sample. However, it should be noted that minor changes in intensity and resolution might occur (see note S3). For each angle, the frequency positions of magnon modes with specific wave numbers were extracted (Fig. 4C). Regarding the fundamental spin wave mode ( n=0 , solid line) we can observe a strong anisotropy in the dispersion relation caused by magnetic dipolar interaction. For the first PSSW ( n=1 , dashed line), where the dipolar interaction is much weaker compared to the exchange interaction, the angular dependence of spin wave frequencies is almost isotropic. The obtained data agree well with the analytical models (*44*, *45*).

In conclusion, we have demonstrated a method for measuring magnons in a previously inaccessible wave vector range using Mie-enhanced microfocused Brillouin light scattering. By integrating periodic dielectric nanostripes into a standard μBLS experiment, we achieved full wave vector resolution, enabling the detection of magnons with wavelengths down to 30 nm (wave vectors up to 200 rad μm^−1^). This technique bridges a long-standing experimental gap, providing opportunities for investigating fundamental spin-wave physics across the entire Brillouin zone or for imaging spin-wave propagation in nanoscale spin-wave devices. Our results establish a robust method for characterizing nanoscale magnons in space, wave number, and frequency with unprecedented resolution, validated through both experiments and theoretical modeling.

Beyond magnonics, the methodology developed here is also applicable to other collective excitations, such as phonons, potentially opening avenues in fields ranging from ultrafast magnetism to mechanobiology (*46*, *47*). These can include, for instance, wave vector resolved measurement of spin currents or of the mechanical properties of tissues and cells in the range of considerably larger wave vector values, which is crucial for modeling across all material parameters. Moreover, this technique could enable the investigation of action potential propagation in a membrane, as it allows measuring acoustic excitations with shorter wavelengths than with standard μBLS. The ability to resolve excitations with nanoscale precision and directional sensitivity could drive further advancements in the scope of wave vector–resolved spectroscopy in condensed matter physics.

## MATERIALS AND METHODS

### Microfocused Brillouin light scattering

Brillouin light scattering is a process in which incident photons are inelastically scattered by quasiparticles such as magnons or phonons, resulting in the creation of photons with different energy and momentum. The process obeys conservation of energy and in-plane momentum, which can be expressed as

$$\omega_{f}=\omega_{i}\pm \omega_{m}$$ (5)

$$k_{f}=k_{i}\pm k_{m}$$ (6)

where $\omega_{i}$ and $k_{i}$ are the angular frequency and in-plane wave vector of the incident photon, $\omega_{f}$ and $k_{f}$ correspond to the scattered photon, and $\omega_{m}$ and $k_{m}$ to the magnon.

In a typical μBLS experiment, the accessible magnon wave vector range in thin-film measurements is determined by the wavelength λ of the probing light and the *NA* of the focusing optics. Together they define the range of incident angles. However, the wave vector range predicted by this geometric consideration does not fully match our experimentally observed detection sensitivity. Instead, the detection is better described by an analytical expression based on the Fourier transform of the beam spot, as discussed in (*10*), and approximated by

$$\text{HWTM}\approx 6.72\frac{\text{NA}}{\lambda }$$ (7)

where *HWTM* is the half-width at 10th maximum of the incident laser intensity in reciprocal space, roughly corresponding to the detection range.

In this paper, we used a custom-developed optical setup to measure Brillouin light scattering spectra (*10*, *36*). A single-mode laser (COBOLT Samba) with a wavelength of 532 nm was used as the light source. The spectral purity of the laser light was improved by a Fabry-Perot filter (TCF-2, Table Stable). The incident power on the sample was 3 mW, which did not introduce any visible nonlinear phenomena or heating of the sample. An optical microscope with active stabilization was used to compensate the mechanical drifts of the sample (THATec Innovation). The light was focused and collected through the same objective (Zeiss LD EC Epiplan-Neofluar 100 ×/0.75 BD). The inelastic frequency shift was measured with a tandem Fabry-Perot interferometer (TFP-2HC interferometer, Table Stable) (*30*). To generate the magnetic field, we used a water-cooled GMW 5403 electromagnet powered by two KEPCO BOP20-20DL power supplies and a predefined current field calibration at the sample position.

### FDTD simulations

Simulations were conducted using the Ansys Lumerical FDTD Solutions software. The simulation region was set to 8000 × 8000 × 1150 nm^3^, with the shortest dimension aligned along the optical axis. The model consisted of a semi-infinite Si substrate coated with a 27.6-nm-thick permalloy film. On top, amorphous Si nanostripes with a periodicity of 150 nm were arranged, covering the entire simulation area. Amorphous Si was selected because of its closer optical resemblance to sputtered Si.

The global mesh was set to conformal variant 0 (mesh order 4), with a refined 2-nm mesh in the central simulation region (1024 × 1024 × 96 nm^3^) in the vicinity of the nanostripes, approximately corresponding to the laser spot size. Perfectly matched layers were used as boundary conditions, and appropriate symmetry conditions were applied. A Gaussian source, modeled using the thin-lens approximation with a *NA* of 0.75, was focused on the top of the center stripe. The source polarization was set either perpendicular or parallel to the nanostripes.

The dielectric functions for the Si substrate, amorphous Si, and permalloy were obtained from (*48*–*50*). Electric field data were collected using a field monitor positioned in the middle of the permalloy film and analyzed with MATLAB 2024a.

### Sample preparation

The sample was fabricated by electron beam lithography and a lift-off process. We started with a Si(100) substrate with a native SiO_2_ layer. A Ni_80_Fe_20_ film (approximately 30 nm thick) was then deposited on the sample by e-beam evaporation at room temperature. For the lithography process, two layers of polymethylmethacrylate resist were spin-coated: a 200-nm layer of Allresist AR-P 649.04 followed by a 60 nm layer of Allresist AR-P 679.02.

Pattern exposure was performed using a RAITH 150-two electron beam writer, followed by development of the exposed resist. Oxygen reactive ion etching was performed to improve adhesion for the subsequent Si deposition. Si was deposited by ion-beam sputtering at room temperature using a crystalline Si target.

**Fig. 5.**
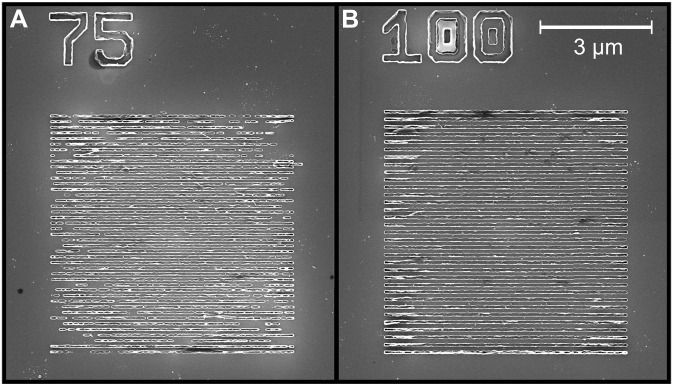
Scanning electron microscope images of fabricated structures. The periodicity is 150 (**A**) and 200 nm (**B**).

The final lift-off process involved immersing the sample in an acetone bath for approximately 24 hours, followed by rinsing with acetone and isopropanol to complete the fabrication. The scanning electron microscope images of nanostripe arrays with periodicities of 150 and 200 nm are shown Fig. 5 (A and B).

### Spectra analysis

The BLS spectra taken on the periodic dielectric nanostripes yields multiple peaks at various frequency positions, which correspond to the different orders of detection or spin wave modes, see Fig. 3B. Therefore, the spectrum needs to be carefully analyzed. First, the frequency positions of all peaks are found and labeled with the corresponding order of detection by periodic array and spin wave mode.

**Fig. 6.**
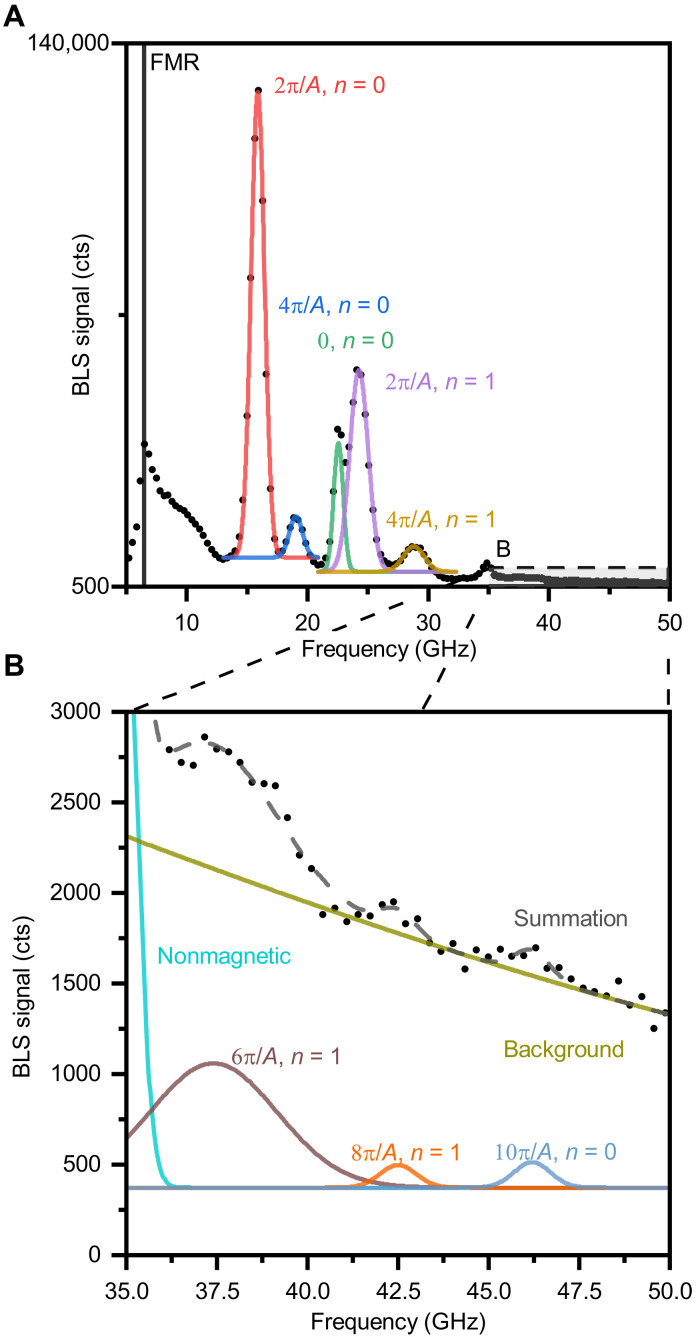
Measured and analyzed BLS spectrum on a grating with A=150 nm. Peaks are fitted with Gaussian curves and matched with corresponding magnon wave numbers. (**A**) shows high signal-to-noise ratio peaks, while (**B**) shows low signal-to-noise ratio peaks.

For well-separated, high signal-to-noise ratio peaks (Fig. 6A), we fit individual Gaussian curve to each peak without any further processing. We used a Gaussian curve instead of a Lorentzian one to model the spectra, because its shape is mainly determined by the Gaussian profile of the probing beam spot.

However, for overlapping low signal-to-noise ratio peaks (above 35 GHz; Fig. 6B), we fit the sum of multiple Gaussian curves and an exponential background to account for quasi-elastic scattering and localized acoustic modes. It is important to note that the spectra in Fig. 6 had a 24-hour acquisition time. The highest measured wave number (approximately 200 rad μm^−1^) has still a signal-to-noise ratio bigger than 3 and thus can be distinguished. We estimate that with substantially shorter acquisition times (tens of minutes), it would not be clearly distinguishable.

After determining peak positions, we proceeded to match them to their corresponding spin wave mode and wave number. A critical consideration in this process is that signals corresponding to lower wave numbers (lower order of detection) typically appear stronger in BLS spectra (this does not apply to ferromagnetic resonance and PSSW at k=0 ). Using this knowledge, we compared the observed peak positions with the predicted dispersion relation of the material to establish frequency–wave number pairs.

This matching process becomes considerably more complex in thicker films due to the emergence of higher-order PSSW modes. This complexity accounts for the gaps present in our final dispersion relation (Fig. 4B). When two different wave numbers, one corresponding to n=0 and another to n=1 , produced overlapping peaks at similar frequencies that could not be clearly distinguished, we attributed the peak to the lower in-plane wave number magnon ( n=1).

### BLS spectra modeling using the reciprocity theorem of electromagnetism

The task of calculating the intensity of the BLS signal collected by a detector can be elegantly completed using the reciprocity theorem (*43*), where we exploit a close link between the fields generated by an electric dipole ( p ) (emulating the polarization current induced within the magnetic layer) and a virtual source located at the position of the detector, see supplemental Fig. 7. Mathematically, this connection can be written down as

$$p_{v}\left(r_{\text{det}}\right)\cdot E_{m}\left(r_{\text{det}}\right)=p_{m}\left(r\right)\cdot E_{v}\left(r\right)$$ (8)

where the subscripts differentiate whether the particular field or dipole moment is associated with the magnetic source (*m*) or the virtual source (*v*). Note that the two dot products are evaluated at different positions in space corresponding to the respective locations of the sources (detector and magnetic sample). The above relation can be translated into a simple statement: If a photon emitted from the detector is able to reach a particular spot on the sample, then it should work also the other way around. Although this is not true in general [e.g., in gyrotropic materials (*51*)], the materials in our sample are linear and isotropic (at least with respect to the out-coupling process that follows the inelastic scattering event) and the reciprocity theorem is valid. While the amplitude of the virtual dipole source can be set arbitrarily, its orientation should match the experimental conditions under which the BLS signal is collected. In presented experiments and calculations, we use a cross-polarized scheme, which is sensitive to the spin wave BLS signal in linear case (*10*). The virtual electric field should capture all the effects arising from the propagation between the detector and the sample, including any interaction with the Si stripes or surrounding media. We assume that the collection and illumination spots are identical with a width of 440 nm and a Gaussian shape (*36*). This is justified, because both branches share the same objective lens and are perfectly aligned. Taking all this information as new settings for our FDTD model, we calculated the distribution of the virtual electric field within the magnetic layer.

**Fig. 7.**
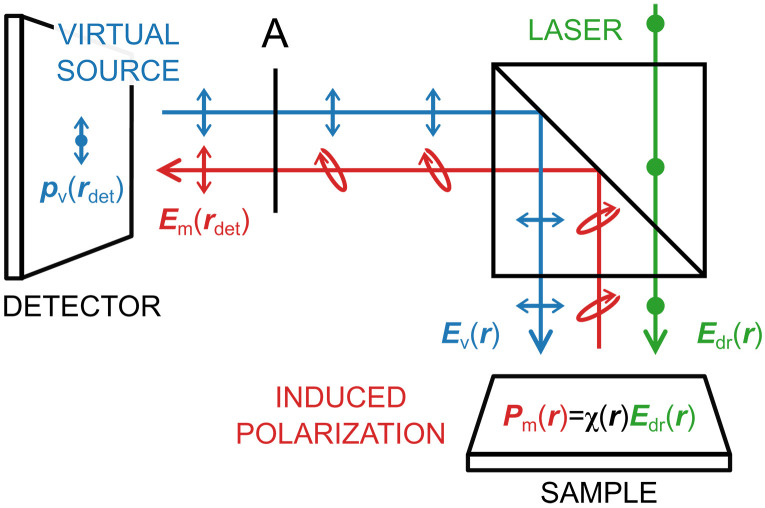
Illustration of the reciprocity theorem concept. It highlights the key physical quantities and their polarization states, parallel, perpendicular, and arbitrary (elliptic). The letter A denotes an analyzer.

To account for the continuous nature of the induced polarization $P_{m}\left(r\right)=\chi \left(r,\omega_{m}\right)E_{\text{dr}}\left(r\right)$ (implicitly assuming linear magneto-optical coupling), the right-hand side of Eq. 8 should be replaced by a volume integral over the magnetic layer

$$\begin{array}{c} p_{v}\left(r_{\text{det}}\right)\cdot E_{m}\left(r_{\text{det}}\right)=\int dr^{3}P_{m}\left(r\right)\cdot E_{v}\left(r\right)=\\ =\sum_{i,j}\int dr^{3}\chi_{m}^{\mathrm{ij}}\left(r\right)E_{v}^{i}\left(r\right)E_{\text{dr}}^{j}\left(r\right) \end{array}$$ (9)

Restricting our analysis to laterally propagating magnons, the magnetic susceptibility tensor can be cast as

$$\chi_{m}\left(r,\omega_{m}\right)=\chi_{m}\left(z,\omega_{m}\right)e^{ir_{m}\cdot r_{∥}}$$ (10)

where $k_{m}$ denotes the magnon wave vector and the symbol ∥ indicates the lateral (in-plane of the magnetic sample) spatial coordinates. The variation of the susceptibility tensor with respect to the vertical coordinate *z* will generally depend on the thickness of the magnetic layer and the PSSW order. Since we are studying metallic layers (NiFe), where the penetration depth of light ( ≈20 nm) is comparable or shorter than the thickness of the sample, we neglect this dependence in our analysis. Inserting Eq. 10 into Eq. 9 and recalling that the detected BLS signal is proportional to the intensity of the collected light, we find the contribution from a single magnon with a wave vector $k_{m}$ to be

$$\begin{array}{c} \begin{array}{c} \sigma \left(k_{m},\omega_{m}\right)∼{\left|p_{v}\left(r_{\text{det}}\right)\cdot E_{m}\left(r_{\text{det}}\right)\right|}^{2}=\\ ={\left|\sum_{i,j}\int dz\chi_{m}^{\mathrm{ij}}\left(z,\omega_{m}\right)\int dr_{∥}^{2}E_{v}^{i}\left(r\right)E_{\text{dr}}^{j}\left(r\right)e^{ik_{m}\cdot r_{∥}}\right|}^{2} \end{array} \end{array}$$ (11)

The above equation indicates that the strength of the BLS signal is largely determined by the overlap integral between the driving and virtual electric field distributions modulated at the frequency of the magnon. It can be perceived as a transfer function of the system that determines the range of detectable magnons and its inspection can be of great value for both the design process and the subsequent analysis of experimental measurements.

## Supplementary Materials

### This PDF file includes:

Note S1 to S3 Figs. S1 to S3
