# Supplementary material for: Mie-enhanced microfocused Brillouin light scattering for full wave vector resolution of nanoscale spin waves
Source: Sci Adv. 2025 Oct 31;11(44):eady8833. doi: 10.1126/sciadv.ady8833 (PMC12577697; doi:10.1126/sciadv.ady8833)
Supplement: Supplementary file 1 — Note S1 to S3 Figs. S1 to S3 [file sciadv.ady8833_sm.pdf]

Science Advances
AAAS

Supplementary Materials for

Mie-enhanced microfocused Brillouin light scattering for full wave vector resolution of nanoscale spin waves

Jakub Krčma *et al.*

Corresponding author: Ondřej Wojewoda, ondrej.wojewoda@vutbr.cz;
Michal Urbánek, michal.urbanek@ceitec.vutbr.cz

Sci. Adv. **11**, eady8833 (2025)
DOI: 10.1126/sciadv.ady8833

This PDF file includes:

Note S1 to S3
Figs. S1 to S3

SUPPLEMENTAL MATERIAL

Supplemental Material Note 1: Thickness determination of the permalloy film via XRR

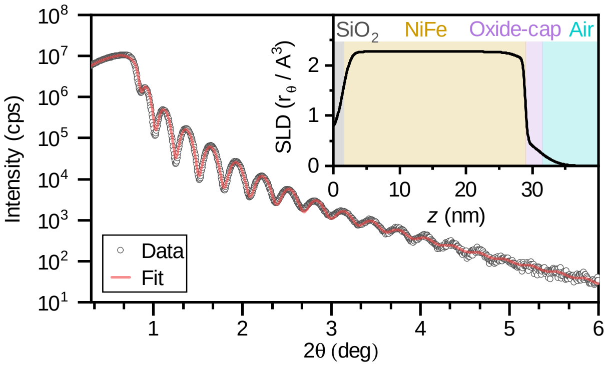

Supplemental Figure S1. **Measured XRR data (open symbols) and corresponding fit (solid line) for the permalloy layer used in the study**. A Si(substrate)/SiO_2/NiFe/oxide-cap multilayer model was employed for the thickness determination of the layers. The inset shows the depth-dependence of the scattering length density (SLD) resulting from the model refinement, where r_θ is the classical electron radius and $z = 0$ corresponds to the Si substrate height.

The thickness of the permalloy film was determined via X-ray reflectivity (XRR) measurements using a Rigaku Smartlab (9 kW) diffractometer with Cu-Kα radiation ($\lambda = 1.54$ Å) and an incident parallel beam setting. Soller slits of 5° in both the incident and diffracted optics were employed. The measured intensity vs. 2θ profile was fitted using the GenX 3 software [52] (see Fig. S1). The multilayer refinement provides for the permalloy film fitted thickness and density values of 27.6 ± 0.1 nm and 8.7 ± 0.1 g/cm^3, respectively. A thin capping layer with a reduced density (~ 30 %) of the permalloy density value) in the model considers the naturally formed oxide on top of the magnetic layer, for which a thickness of 2.5 ± 0.5 nm is obtained.

Supplemental Material Note 2: Robustness of optical response across grating periods

To verify that our Si gratings represent a robust platform for probing magnons, we simulated (FDTD) their optical response across a range of grating periods (from 150 nm to 300 nm) in terms of their scattering cross-section. More specifically, we evaluated the power scattered by the gratings under plane wave illumination with polarizations both perpendicular (Fig. S2A) and parallel (Fig. S2B) to the grating at normal incidence over an area of 5 µm × 5 µm. Taking into account both configurations is crucial as one polarization is relevant during illumination while the other during the collection of the light, see Fig. 7. The resulting spectra shown in Fig. S2 demonstrate that at the operation wavelength (532 nm) and close to its vicinity, the response is flat and free of any sharp features for all the grating periods. As such, Si gratings can be considered a robust system well suited for BLS measurements.

While the scattering cross-section gives us a qualitative insight into how the gratings will perform in BLS experiments, a more quantitative estimation—taking into account, e.g., the finite NA of the objective—should be based on the reciprocity theorem and the transfer matrices $T_{ij}(\boldsymbol{k}_\mathrm{m} z)$ defined by Eq. 4.

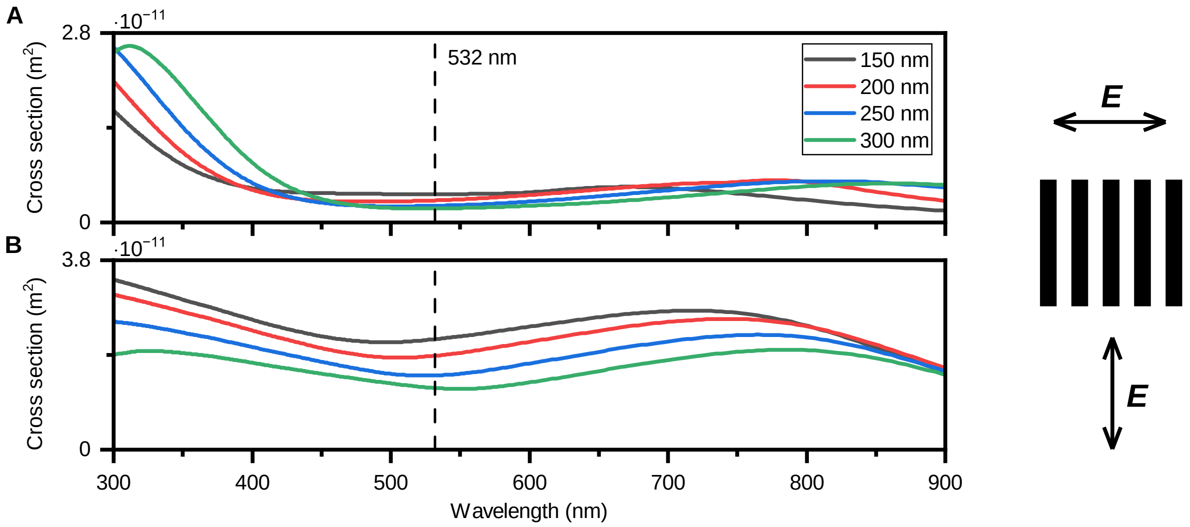

Supplemental Figure S2. **FDTD simulated scattering cross-section spectra of Si gratings with different grating periods**: (**A**) for polarization perpendicular to the grating and (**B**) for polarization parallel to the grating. Their flat optical response around the BLS operation wavelength indicates that Si gratings are well suited for expanding the measurement range of magnon k-vectors.

Supplemental Material Note 3: Directional sensitivity of the transfer matrices

To investigate how the one-dimensional periodicity of the nanostripes imposes directional selectivity on the BLS process, we calculated the transfer function components for three stripe orientations (0°, 45°, 90°) with respect to the fixed incident polarization.

As shown in Fig. S3, the matrix components reveal that for the 0° and 90° orientations, the detection peaks appear strictly perpendicular to the nanostripe direction. The in-plane magnetization components effectively swap between these orientations, which also impacts the final BLS signal strength. For instance, when the external field is applied along the y-direction, the static magnetization aligns with it, and the spin-wave precession occurs in the perpendicular x, z-plane. As a result, there is no dynamic magnetization along y, rendering the corresponding component of the transfer function irrelevant to the measured BLS signal. In our case, this leads to a reduced signal in the 0° orientation, where the transfer function shows higher sensitivity for the y-components. In contrast, in the 90° orientation, where the there is higher sensitivity for x-components, the signal is stronger. This indicates that, for stripe arrays, it is advantageous to apply the external field perpendicular to the stripe orientation to maximize the signal strength.

In the case of 45°, there is onset of the non-k specific background, but the prominent peaks and the positions of the periodicity of the nanostripes still allow the k-vector analysis. Note, that in this case we are sensitive also to the perpendicular direction.

The directional confinement persists regardless of nanostripe–polarization axis angle, confirming that the periodic modulation of the near field alone defines the angular sensitivity. These results demonstrate that Mie–enhanced µBLS via periodic dielectric arrays provides robust, orientation-tunable wavevector resolution without requiring changes to the optical geometry.

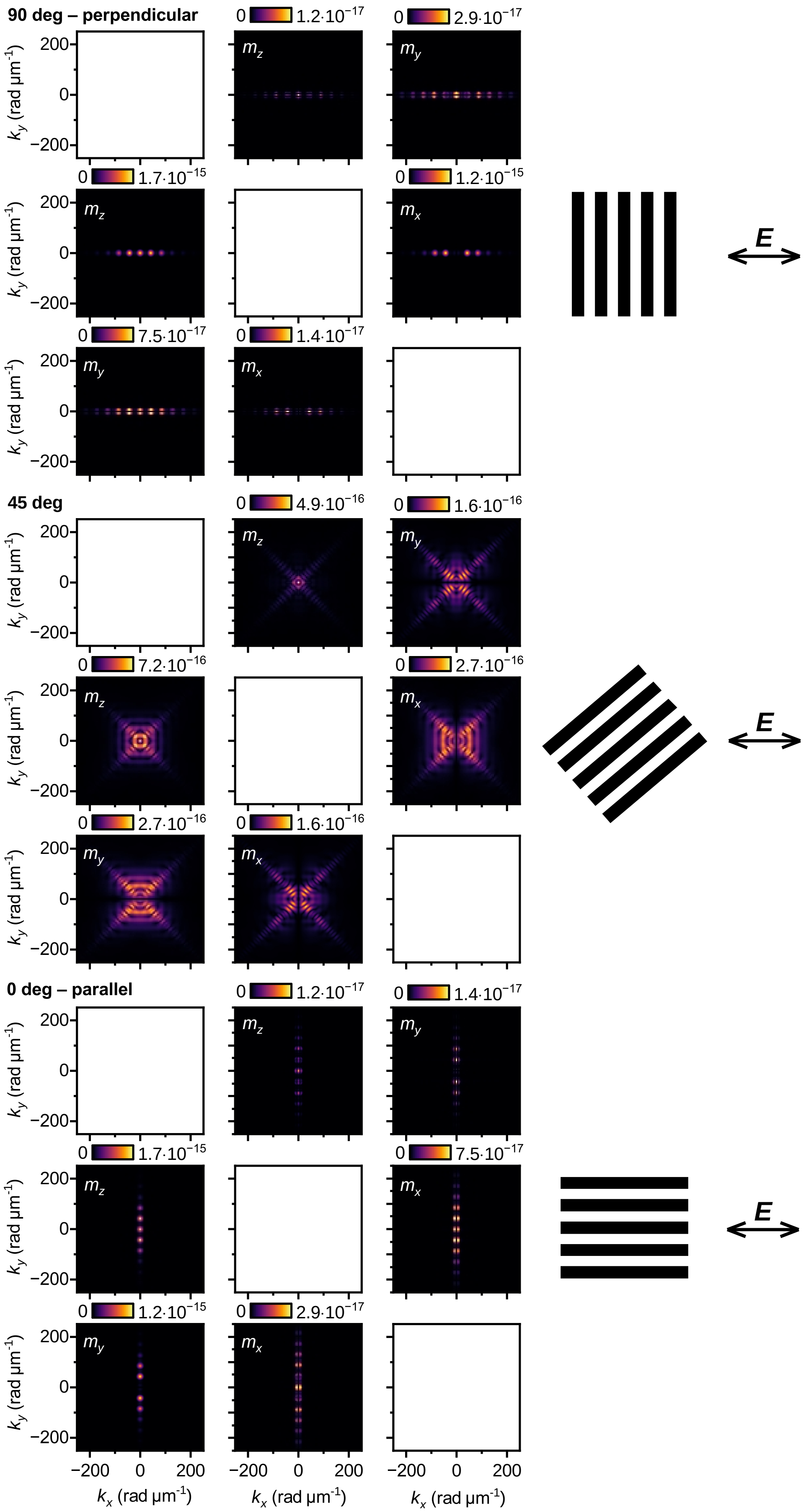

Supplemental Figure S3. **Transfer functions for different nanostripe–polarization axis angles**. The period of the nanostripes is $A = 150\,\mathrm{nm}$.
